# Myocardial strain analysis in patients with Heart Failure with preserved Ejection Fraction using bright blood cine MR images: A comparison with speckle-tracking echocardiography

**DOI:** 10.1186/1532-429X-16-S1-P71

**Published:** 2014-01-16

**Authors:** Peter M Smith, Vistasp Daruwalla, Benjamin H Freed, Bruce S Spottiswoode, Kevin Kalisz, James C Carr, Jeremy D Collins

**Affiliations:** 1 Department of Radiology, Northwestern University, Chicago, Illinois, USA; 2 Cardiovascular MR R&D, Siemens Healthcare, Chicago, Illinois, USA; 3 Department of Cardiology, Northwestern University, Chicago, Illinois, USA

## Background

Changes in myocardial strain parameters is of interest in patients with heart failure as an objective measure of disease severity. Speckle-tracking echocardiography (ST-echo) is the accepted standard of reference for myocardial strain analysis given superior temporal resolution; however, difficult acoustic windows and limited contrast to noise resolution can limit strain analysis. Preliminary work using deformation field analysis at steady state free precession (SSFP) cine MR imaging has shown that strain analysis at CMR is similar between conventional and highly accelerated GRAPPA cine acquisitions. The purpose of this study is to compare the strain values in patients with heart failure and preserved ejection fraction (HFpEF, left ventricular ejection fraction >50%) at SSFP cine MRI with ST-echo.

## Methods

Retrospective analysis of Cardiac MR and echocardiographic images from 15 patients (5 men, avg age 61.2 yrs) with HFpEF. Cardiac MR images were obtained at 1.5 T (MAGNETOM Avanto, Siemens Medical Systems, Erlangen, AG) using GRAPPA factor 2 acceleration (temp res = 39.2 msec, spatial res = 1.5 × 1.5 mm, thickness = 6 mm). Myocardial strain analysis at Cardiac MR was performed using prototype software calculating Lagrangian strain from deformation field analysis (Siemens Corp, Corporate Technology, Princeton, NJ). Transthoracic echocardiography exams included apical 4-chamber and mid-ventricular short axis views. Left ventricular (LV) mid ventricular average and peak systolic radial and circumferential strains as well as longitudinal strain data was calculated. Peak and average right ventricular (RV) longitudinal strain was also obtained. CMR and ST-Echo derived strain indices were compared using the Pearson correlation. Inter and intraobserver variance was assessed for CMR-derived RV and LV longitudinal strain analysis using the intraclass correlation coefficient (ICC).

## Results

**Figure 1:**
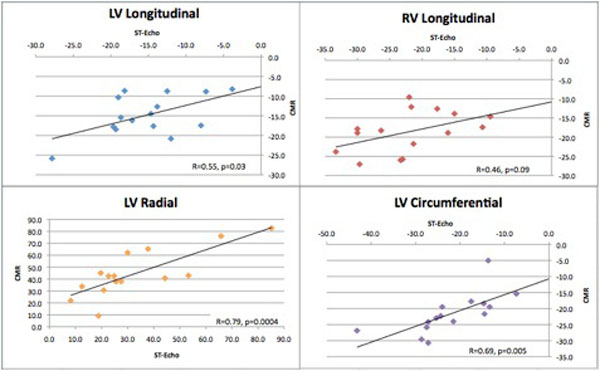
**Correlation plots comparing CMR determined Lagrangian strains with those at ST-echo**. ST-echo: speckle-tracking echocardiography, CMR: Cardiac MR.

Myocardial strain analysis was feasible in all patients with both imaging modalities. There was moderate-strong correlation between CMR and ST-Echo (Figure 1) as follows: LV radial strain (R = 0.79, p = 0.0004), LV circumferential strain (R = 0.69, p = 0.005), and LV longitudinal strain (R = 0.55, p = 0.03). Fair correlation was noted for RV longitudinal strain (R = 0.46, p = 0.09). Both inter- and intraobserver reproducibility was excellent (Table 1).

**Table 1:** ICC values demonstrating reproducibility of LV and RV longitudinal CMR strain analysis.

	ICC	95% Confidence Interval
LV Longitudinal	0.997	0.999-0.990

RV Longitudinal	0.981	0.993-0.944

## Conclusions

Although there is a good correlation between CMR derived and ST-echo derived strain analysis in HFpEF patients, the two modalities are not interchangeable and greater standardization is required. Ongoing work using accelerated high temporal resolution SSFP CMR cine acquisitions suggests improved peak strain resolution, which may improve agreement with ST-echo.

## Funding

None.

